# Comparing environmental footprints of haemodialysis and online haemodiafiltration in Italy

**DOI:** 10.1007/s11255-026-05033-3

**Published:** 2026-02-03

**Authors:** James Larkin, Giulia Ligabue, Niccolo Morisi, Gaetano Alfano, Rodrigo Martínez-Cadenas, Abass Fehintola, Ingeborg Steinbach, Aycan Yasar, Marta Arias-Guillén, Francesc Maduell Canals, Karin G. F. Gerritsen, Francis Mortimer, Gabriele Donati, Brett Duane

**Affiliations:** 1 School of Dental, Child and Public Health, Trinity College Dublin, Dublin, Ireland; 2 Surgical, Medical and Dental Department of Morphological Sciences Related to Transplant, Oncology and Regenerative Medicine, Section of Nephrology, University of Modena and Reggio Emilia, Modena, Italy; 3 Universidad Autonoma de Madrid, Madrid, Spain; 4 Centre for Sustainable Healthcare, Oxford, UK; 5 Hospital Clinic de Barcelona, Barcelona, Spain; 6 University Medical Center, Utrecht, The Netherlands

**Keywords:** Haemodialysis, Haemodiafiltration, Life cycle assessment, Environmental impact, Carbon footprint, Energy use, Water consumption, Sustainable kidney care

## Abstract

**Background:**

Haemodialysis (HD) and online haemodiafiltration (OLHDF) are the main in-centre treatments for kidney failure. Both rely on high water and energy use and produce substantial greenhouse gas emissions. OLHDF provides superior solute clearance and improved survival compared with high-flux HD, but its environmental burden remains less defined. Clarifying these differences supports evidence-based and sustainable treatment decisions.

**Methods:**

A process-based life cycle assessment (LCA) was performed at the Nephrology, Dialysis and Kidney Transplant Unit, AOU Policlinico di Modena, Italy, in 2024, following ISO 14040 and 14,044 standards. The functional unit was one patient year of treatment, equal to 156 sessions. System boundaries included procurement, water treatment, session operations, travel and waste management. Modelling used OpenLCA with Ecoinvent 3.11 and the Italian electricity grid factor of 0.25 kg CO2 per kWh. Scenarios assessed HD-only, OLHDF-only and the real-world Modena treatment mix. Sensitivity analysis varied the share of OLHDF, session frequency, grid intensity and reverse-osmosis (RO) recovery rate and included a reduced-flow OLHDF prescription.

**Results:**

The annual footprint was 4469 kg CO2-eq, 60,290 MJ and 1364 m3 world-eq deprived water per patient year. HD generated 4427 kg CO2-eq and OLHDF 4548 kg CO2-eq, reflecting slightly higher electricity and water consumption and greater plastic use in OLHDF. Travel contributed 71% of total emissions and procurement 21%. Sensitivity analysis showed changes in RO efficiency and electricity mix had stronger effects than treatment type.

**Conclusions:**

HD and OLHDF have comparable environmental profiles. Clinical outcomes should drive modality choice, while sustainability gains depend on improving transport, water recovery, energy management and renewable integration.

**Supplementary Information:**

The online version contains supplementary material available at 10.1007/s11255-026-05033-3.

## Introduction

Chronic kidney disease (CKD) affects more than 850 million people globally, making it a major public health challenge [1]. Many patients progress to kidney failure requiring kidney replacement therapy (KRT) in the form of transplantation or dialysis [2]. Haemodialysis (HD) remains the most widely used KRT, delivered primarily in hospital or satellite centres. Online haemodiafiltration (OLHDF), which combines diffusive and convective clearance, is increasingly adopted because of its clinical benefits, including improved survival compared with high-flux HD [3].

Dialysis is among the most resource-intensive areas of healthcare. A single HD session typically consumes up to 370–600 L of water and 5–15 kWh of electric energy from optimal to conventional settings [4–6]. This equates to tens of thousands of litres of water and thousands of kilowatt-hours per patient each year. In one Australian study, dialysis services were estimated to account for up to 7% of total hospital carbon emissions, driven primarily by patient transport, electricity use, water consumption and single use consumables. This figure, derived for a specific high income hospital context, provides an order of magnitude indication of dialysis-related impacts rather than a centre specific estimate [7]. Such figures are particularly concerning in light of international commitments to healthcare decarbonisation, including the European Green Deal and net-zero targets set by health systems, such as the NHS [8, 9].

Life cycle assessment (LCA) provides a systematic framework to evaluate environmental impacts from cradle to grave, covering resource extraction, manufacturing, transport, use and disposal [10, 11]. LCA has been applied to healthcare in several contexts, including surgical equipment, imaging and pharmaceuticals [12, 13]. In nephrology, it has been used to map peritoneal dialysis (PD) pathways, HD consumables and entire treatment pathways [14–16].

Quantifying the environmental impacts of medical treatments can support decarbonisation policies, procurement and technology development by identifying hotspots and enabling evidence-based mitigation strategies. At the same time, disclosing environmental performance carries a risk of misinterpretation or inappropriate trade-offs if environmental indicators are considered in isolation from clinical outcomes, equity and patient preferences. Incorporating LCA findings into kidney care, therefore, requires careful communication and integration with broader quality and safety goals [12].

In dialysis LCAs, system boundaries are typically defined from cradle to grave, encompassing production and transport of consumables, water treatment, session operations and waste management, with or without patient transport depending on data availability. Functional units often use a patient year or single dialysis session as a declared unit of service, allowing comparison across modalities while recognising that clinical outcomes, such as survival and hospitalisation rates, may differ between treatments. This study adopts this convention while explicitly acknowledging its limitations for comparing modalities with unequal effectiveness [12, 13].

Clinically, OLHDF has been shown to improve outcomes. The CONVINCE trial demonstrated reduced mortality and higher quality-adjusted life years compared with high-flux HD, while a pooled analysis of four large randomised controlled trials comparing HD and OLHDF also showed improved overall and cardiovascular mortality with OLHDF [3], [17, 18]. Cost–utility analysis suggests these benefits come at higher financial cost, largely due to extended survival [19]. Environmentally, however, OLHDF requires additional resources, including higher water use and dedicated tubing sets with integrated substitution lines that increase plastic consumption. This indicates that while HDF may be clinically superior, it is likely more resource intensive.

This study aimed to compare HD and OLHDF using detailed LCA based on primary data from the Nephrology Dialysis and Kidney Transplant Unit of the Policlinico di Modena, Italy. Objectives were to (a) quantify the total HD pathway footprint, (b) calculate separate footprints for HD and OLHDF and (c) present weighted results reflecting real-world treatment distribution. Findings are intended to inform clinical decision-making, policy and technology development in sustainable kidney care. This paper is part of the KitNewCare project, which seeks to reduce the environmental impact of kidney care through sustainable innovations [20].

## Methods

### Goal and scope definition

In line with ISO 14040/44, the Methods are organised into goal and scope definition, life cycle inventory and life cycle impact assessment.

The functional unit was defined as one patient year of in‑centre dialysis, equivalent to 156 sessions, reflecting a typical thrice‑weekly treatment schedule used in routine practice and previous dialysis LCAs. Functionally, this represents the provision of kidney replacement therapy for one patient over 1 year in an in‑centre setting, including all associated consumables, water treatment, session operations, travel and waste management. Because OLHDF improves clinical outcomes relative to high‑flux HD, this patient year is treated as a declared unit for comparative environmental profiling rather than a strictly functionally equivalent unit, and the limitations of this approach are discussed in the Discussion [21].

A cradle-to-grave boundary was applied, covering:

1. Procurement: production and inbound transport of dialysers, bloodlines, concentrates, needles and other single‑use consumables, including associated plastics, packaging and manufacturing energy.
2. Water treatment: At the Modena centre, feed water is municipal tap water, treated on-site by an RO system to produce ultrapure product water for HD and OLHDF; no separate deionised water system is used. Supply of municipal tap water, on‑site reverse osmosis (RO) treatment and distribution loops to generate ultrapure product water, including electricity use and discharge of reject water. Data on the disinfection of internal water distribution pipelines were not available and were, therefore, not included; this omission is expected to have minimal influence on total impacts.
3. Session operations: electricity use of Fresenius 5008 machines, dialysate preparation and substitution fluid generation for OLHDF, and ancillary processes during treatment (including standby and thermal disinfection cycles).
4. Waste management: segregation, collection, transport and treatment (autoclaving, incineration, landfill) of hazardous and non‑hazardous solid and liquid wastes from dialysis sessions.
5. Travel: patient and staff commuting to and from the dialysis centre, modelled as round‑trip journeys per session.

**Fig. 1:**
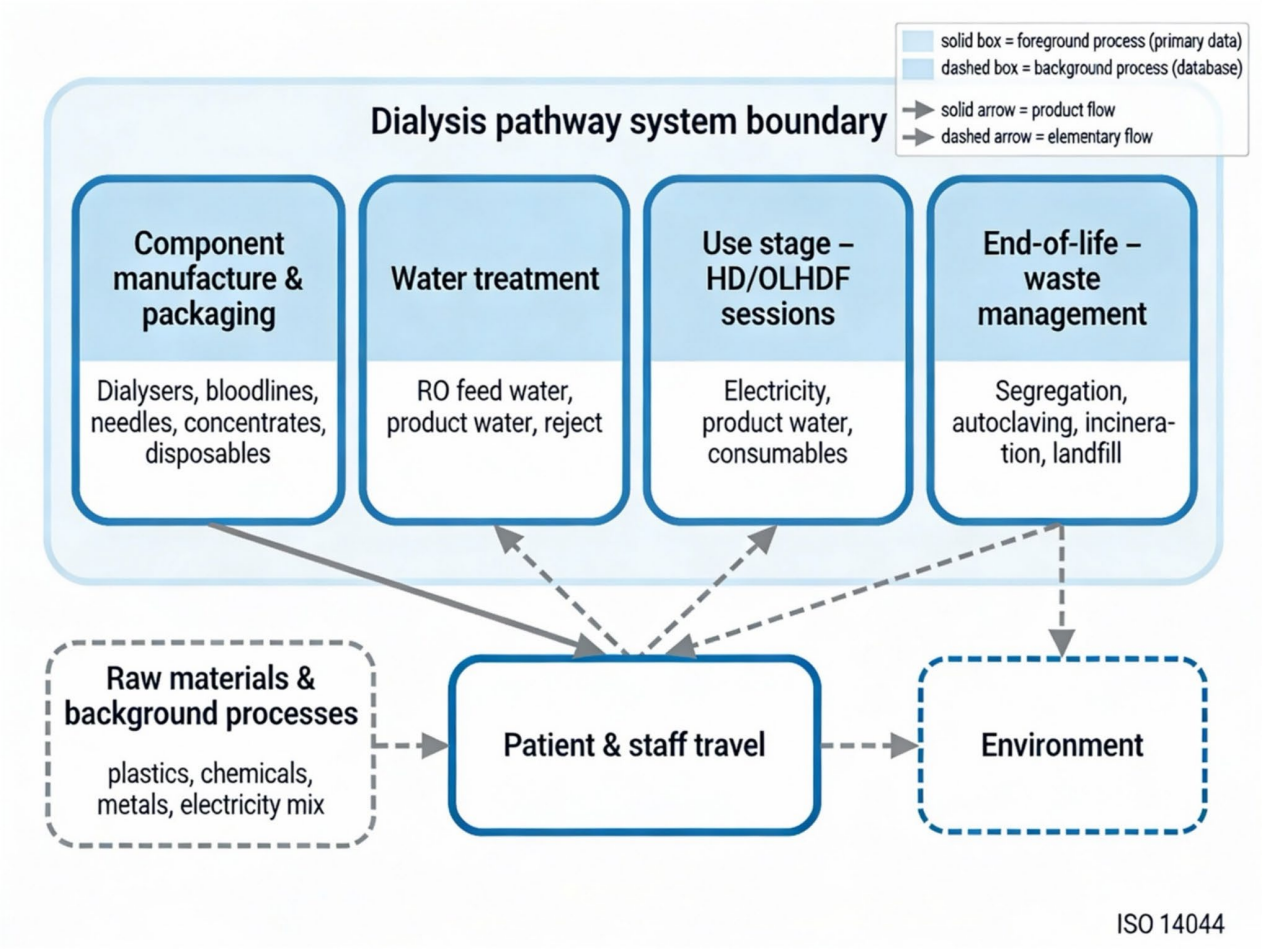
System boundary (ISO 14040/44 aligned). Upstream: raw materials, component manufacturing, packaging and inbound transport for dialysers, bloodlines, concentrates and other consumables. Core/use: municipal tap water supply and RO treatment, electricity use of Fresenius 5008 machines, dialysate and substitution fluid preparation, and use‑phase consumables. Downstream: segregation, collection, transport and treatment of solid and liquid wastes, and patient and staff travel to and from the dialysis centre. Foreground processes (primary data from Modena) are shown as solid boxes; background processes (Ecoinvent 3.11) are shown as dashed boxes

System boundaries included procurement, water treatment, session operations, travel and waste management [12, 13]. The analysis excluded pharmaceuticals, vascular access creation and maintenance, upstream building and equipment infrastructure and other hospital services outside the dialysis unit. These elements were omitted due to limited data and an expected relatively small contribution at the level of a single centre; their exclusion is noted as a limitation Fig. 1.

### Life cycle inventory

#### Procurement

Procurement data were obtained from hospital purchasing records and supplier specifications. Each consumable was dismantled and weighed to determine material composition, similar to approaches used in previous dialysis LCAs [14, 15].

#### Energy

Energy consumption was directly measured for both HD and OLHDF during active treatment and disinfection cycles using a calibrated plug-in energy meter connected to the Fresenius 5008 machines. Measurements covered the full 4-h treatment duration plus thermal disinfection and were repeated across multiple sessions for each modality; average per-session values were then derived. These data replaced previous assumptions of negligible difference between modalities. Energy use included active treatment and standby modes.

#### Water

Water use was measured at the on-site RO system using a dedicated flow meter installed on the product water line. Volumes were recorded over the data collection period and averaged per session, distinguishing HD and OLHDF based on the number of sessions delivered for each modality. Operational water requirements were, therefore, assigned as 382 L per session for HD and 409 L for OLHDF, with values cross-checked against published data for similar RO systems [4].

#### Waste

Waste management practices were confirmed with the hospital’s waste contractor, including segregation into hazardous and non-hazardous streams, autoclaving, incineration and landfill.

All underlying data sets and product images used for this analysis are openly available through Zenodo and referenced in the Supplementary Material.

#### Transport

Patient and staff travel were included in the system boundaries. Patient round trip distances per session were derived from an Irish HD cohort study, because equivalent patient level transport data were not available for Modena; the distribution of dialysis centres and reliance on in centre treatment are broadly similar in both settings, making these data a reasonable proxy for average travel distances. Staff commuting was modelled using average round-trip distance and car use from national statistics. Travel was represented using a mix of passenger car transport processes from Ecoinvent 3.11, reflecting the vehicle types and fuel classes documented in the supplementary modelling workbook [29].

A summary table distinguishing primary measured data, secondary literature-based parameters and background Ecoinvent data sets for key inventory items is provided in the Supplementary Material and referenced in Appendix 1.

### Life cycle impact assessment

At Modena, the treatment mix was 52% HD, 47% OLHDF and 1% Haemodiafiltration with online reinfusion of the endogenous ultrafiltrate (HFR). HFR was excluded due to negligible use and not the purpose of this study. Scenario modelling was conducted for HD-only, OLHDF-only and the weighted Modena mix.

All inventories were modelled in OpenLCA version 2.5.0 using the Ecoinvent 3.11 database [22]. LCIA was performed using the EF v 3.1 midpoint method without normalisation or weighting. Background processes included European data sets for plastics, transport and electricity generation. The Italian electricity grid was modelled at an average carbon intensity of 0.25 kg CO₂/kWh [23].

Machine production was excluded, consistent with conventions in healthcare LCAs [12],[24]. Previous work has shown that capital equipment typically contributes < 5% of the footprint of high-intensity therapies [25].

Three midpoint categories were reported:

- Climate change: kg CO₂ equivalents,
- Energy resources: MJ, net calorific value,
- Water use: m^3^ world eq deprived.

Operational litres were reported alongside LCA water scarcity values to ensure transparency, since LCIA water metrics do not directly reflect volumetric use [26].

Climate change, water use and energy resources were selected as impact categories, because they represent the most material environmental concerns for dialysis care. Climate change is the most widely reported and policy-relevant endpoint in healthcare LCAs, allowing comparability across studies and alignment with decarbonisation targets [10], [12]. Water use was included due to the high operational volumes required for dialysate preparation and the growing recognition of water scarcity as a critical planetary boundary [26]. Energy resources were reported, because electricity use is a major contributor to dialysis impacts, with results sensitive to regional grid intensities [23]. Other categories such as toxicity, land use and resource depletion were not prioritised, as prior studies have shown these to be relatively minor contributors in dialysis pathways compared with carbon, water and energy [14, 15].

Accordingly, this study is presented as a process‑based life cycle assessment focusing on three key midpoint categories (climate change, energy resources, water use). Other impact pathways (e.g., toxicity, land use, and resource depletion) were not quantified and their omission is acknowledged as a limitation. The reported climate change results do not constitute a standalone ISO 14067 carbon footprint study.

Differences between HD and OLHDF were modelled as follows:

- Water: HD consumes 382 L per session, OLHDF 409 L, based on measured substitution volumes [4]. Blood flow rates of approximately 350 mL/min were applied as well as a convective volume of at least 23L/session [27]. RO recovery was set at 65%, meaning the 35% of feed water is discharged as reject [28].
- Procurement: Both modalities used the Fresenius 5008 machine. OLHDF required two dialysate-side ultrafilters for online substitution fluid generation, replaced quarterly as per manufacturer guidance, while HD did not use these additional filters. The tubing set for OLHDF included integrated substitution lines, resulting in higher plastic mass and associated emissions.

Foreground flows were matched to Ecoinvent 3.11 processes based on technology similarity and regional scope (European or global averages, where Italian data were not available), following cutoff allocation. A mapping table listing the main foreground flows (e.g., dialyser plastic types, tubing sets, electricity, transport, and waste treatments) and their associated Ecoinvent processes is provided in the Supplementary Material Appendix 1 to support transparency and reproducibility.

Sensitivity analysis examined the influence of operational and external parameters on total results. Variables tested included:

- (1) The proportion of OLHDF treatments (0–100%),
- (2) Treatment frequency (150–160 sessions per year),
- (3) Electricity grid carbon intensity (Italy 0.25 vs EU average 0.29 kg CO₂/kWh) and
- (4) RO recovery efficiency (50–80%).

A reduced-flow OLHDF scenario was also modelled using a Qd/Qb (dialysate to blood flow) ratio of 1.2 and a convective volume of 25 L, consistent with literature-reported water-saving prescriptions. Directly measured energy and water values replaced earlier assumptions of equivalence between modalities. For each scenario, total annual results were recalculated and compared with the baseline in absolute terms and as percentage changes to facilitate interpretation.​Complete results are presented in Appendix 2.

Ethical approval was not required for the project detailed in the document, because it involved the environmental assessment of procurement processes and medical devices, rather than research on human participants, patient data, or any other ethical domain requiring institutional review board (IRB) oversight. The focus was on analysing the lifecycle and environmental impacts of products, which does not typically intersect with areas necessitating formal ethical considerations.

## Results

### Total haemodialysis pathway

**Fig. 2:**
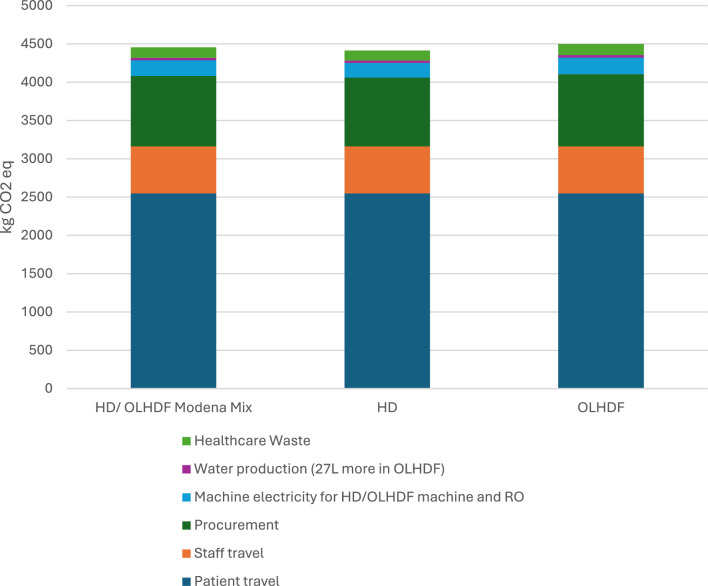
Annual climate impact (kg CO_2_ -eq) of the full HD pathway for one patient year at Modena (52%, 47% OLHDF), including patient and staff travel, procurement, water treatment, session operations and waste management. Bars show contributions by life cycle stage

The full HD pathway at the Nephrology Dialysis and Kidney Transplant Unit of the AOU Policlinico di Modena produced an annual environmental footprint of 4,469 kg CO₂-eq, 60,136 MJ energy resources and 1,361 m^3^ world eq deprived water per patient year (Fig. 2).

The water use indicator (m^3^ world‑eq deprived) expresses potential deprivation of water availability for other users in water‑scarce regions, and, therefore, does not equal volumetric water consumption; both scarcity‑weighted results and measured litres are reported for transparency.

### Category contributions

The largest contributor to emissions was patient travel, responsible for 2,548 kg CO₂-eq, or about 57% of the total pathway footprint. Staff travel contributed 615 kg CO₂-eq. (14%), representing commuting by clinical staff working in the dialysis unit. These figures were based on an average round-trip distance of 29 km per session, derived from Irish haemodialysis travel data, as equivalent Italian patient-level data were not available for ethical reasons as equivalent Italian patient level data were not available for ethical reasons; this proxy influences absolute footprint values but does not affect the relative comparison between HD and OLHDF, because both modalities share the same travel assumptions. The travel distance is considered representative of typical in-centre dialysis access patterns in Modena [29].

Procurement generated 919 kg CO₂-eq in the combined pathway, with 896 kg CO₂-eq for HD and 942 kg CO₂-eq for OLHDF. The higher value for OLHDF reflects the use of a dedicated tubing set with an integrated substitution line, which increases the quantity of plastic and packaging compared with the standard HD bloodline set. OLHDF also requires two additional ultrafilters within the machine, replaced quarterly, which contribute a negligible increase of approximately 0.00011 kg CO₂-eq per patient year to the total footprint.

Electricity use, including the dialysis machine and RO system, accounted for 192 kg CO₂-eq for HD and 215 kg CO₂-eq for OLHDF. Machine electricity, including both the dialysis machine (2.1 kwh per patient per session for HD) and RO systems (3.1 kwh per patient per session) were taken. Direct measurements also showed that OLHDF consumed approximately 5.7% more electricity than HD, mainly due to the additional substitution pump and convective flow operation.

Water production contributed 32 kg CO₂-eq for HD and 35 kg CO₂-eq for OLHDF, consistent with the additional 125 L of ultrapure water required per session to generate substitution fluid. Healthcare waste accounted for 130 kg CO₂-eq for HD and 143 kg CO₂-eq for OLHDF, again reflecting the larger volume of plastic consumables used in OLHDF. The residual “Other” category contributed 14 kg CO₂-eq (< 1%), covering supporting hospital services, such as lighting, cleaning and administration.

### Session contributions and modality comparison

Environmental burdens at the session level were similar between modalities, with only small differences arising from higher material use, energy demand and water consumption in OLHDF.

• HD only: 4,427 kg CO₂-eq per patient year (28.38 kg CO₂-eq per session).

• OLHDF only: 4,548 kg CO₂-eq per patient year (29.16 kg CO₂-eq per session), reflecting the additional electricity required for substitution pumping, slightly greater water use and the higher material demand from HDF-specific tubing and waste.

• Weighted mix (centre practice): 4,469 kg CO₂-eq, 60,290 MJ and 1,364 m^3^ world-eq deprived water per patient year.

Per-session values for the weighted Modena mix were 28.6 kg CO₂-eq, 386 MJ and 8.7 m^3^ world-eq water Table 1.

**Table 1:** Annual environmental footprints of HD, HDF and weighted mix

Scenario	Carbon (kg CO₂-eq/year)	Energy (MJ/year)	Water (m^3^ world-eq/year)
Full pathway (weighted mix)	4469	60,290	1,364
HD only	4,427	58,900	1,364
OLHDF only	4,548	62,250	1,366

Minor differences in total impacts were mainly due to higher procurement, electricity and water use in OLHDF. The overall variation between modalities remained below 3%, showing that treatment type has minimal influence on total pathway emissions.

To isolate the effects of the dialysis technologies and facility operations, we also calculated results excluding patient and staff travel. Without transport, annual climate impacts were 1,306 kg CO₂‑eq for HD and 1,427 kg CO₂‑eq for OLHDF, a 9.3% difference driven mainly by higher material use, water consumption and electricity demand in OLHDF. This confirms that travel assumptions strongly affect absolute pathway footprints but do not materially alter the relative comparison between modalities.

The water use indicator (m^3^ world‑eq deprived) expresses potential deprivation of water availability for other users in water‑scarce regions, and, therefore, does not equal volumetric water consumption; both scarcity‑weighted results and measured litres are reported for transparency.

### Sensitivity analysis

A sensitivity analysis was performed to assess the effect of treatment modality and operational parameters on total environmental results. The baseline scenario represented one patient year of in-centre dialysis at AOU Policlinico di Modena (52% HD, 47% OLHDF, 156 sessions, Italian grid factor 0.25 kg CO₂/kWh).

Conventional HD produced 4,427 kg CO₂-eq per patient year, while standard high-volume OLHDF reached 4,548 kg CO₂-eq, a 2.7% increase due to higher consumable use, 27 L more ultrapure water per session and 5.7% higher electricity consumption.

A reduced-flow OLHDF scenario was modelled using a Qd/Qb ratio of 1.2 and a convective flow of 25 L [30]. This configuration reduced dialysate flow from 125 to 100 L per session, saving about 25 L of water and reducing carbon emissions by 3–4 kg CO₂-eq per patient year while maintaining a similar Kt/V (urea clearance time volume index).

Varying the share of OLHDF from 0 to 100% changed total emissions by less than 3%. Adjusting the number of sessions between 150 and 160 shifted results by under 3%. Applying the EU-average grid factor (0.29 kg CO₂/kWh) increased total emissions by 0.7%.

Improving RO recovery from 50 to 70% reduced total feed and reject water volumes by roughly 30%. For HD, feed water decreased from 119 m^3^ to 85 m^3^ and reject water from 59.6 m^3^ to 25.6 m^3^ per patient year. For OLHDF, feed water decreased from 128 m^3^ to 91 m^3^ and reject water from 63.8 m^3^ to 27.3 m^3^. The carbon saving from this improvement was approximately 3–4 kg CO₂-eq per patient year Table 2.

**Table 2:** Sensitivity analysis results

Scenario	Carbon (kg CO₂-eq/year)	Energy (MJ/year)	LCIA water (m^3^ world-eq/year)	Notes
Baseline (52% HD, 47% OLHDF, Italy grid)	4469	60 290	1364	Full pathway
0% OLHDF (HD only)	4427	58 900	1364	All HD
75% OLHDF	4509	61 700	1365	Higher HDF share
100% OLHDF	4548	62 250	1366	All HDF
150 sessions (HD only)	4270	57 940	1312	Fewer sessions
160 sessions (HD only)	4555	61 730	1384	More sessions
EU grid factor (0.29 kg CO₂/kWh)	4500	n/a	n/a	Approximately 0.7% CO₂ increase vs baseline
RO recovery 50%	n/a	n/a	HD feed 119 m^3^/reject 59.6 m^3^; HDF feed 128 m^3^/reject 63.8 m^3^	Less efficient RO
RO recovery 70%	n/a	n/a	HD feed 85 m^3^/reject 25.6 m^3^; HDF feed 91 m^3^/reject 27.3 m^3^	30% less reject water
Reduced-flow HDF (100 L dialysate, 25 L convective)	4515	60 290	1339	25 L less water per session; 3–4 kg CO₂-eq reduction per year

According to Bendine et al. (2020), total water use per dialysis session, including disinfection, is about 300 L using the same RO system as Modena, indicating that roughly 175 L of water are consumed for disinfection outside the treatment cycle [4].

## Discussion

This study quantified the environmental impacts of HD and OLHDF using a process-based life cycle assessment based on primary data from a large Italian centre. The reconciled annual footprint of the dialysis pathway was 4,469 kg CO₂-eq, 60,290 MJ and 1,364 m^3^ world-eq deprived water per patient year. This provides a benchmark for the environmental burden associated with in-centre kidney replacement therapy, reflecting real-world clinical practice.

Patient and staff travel were the dominant contributors, together accounting for about 71% of total emissions. Transport not only represents the largest environmental burden but also a major economic cost in kidney care, estimated to account for roughly half of total dialysis expenditure in some healthcare systems, including the United States, where annual transportation costs for in-centre dialysis have been reported at about 3 billion USD [31].

At the session level, HD and OLHDF showed almost identical results. Per-session impacts were 28.7 kg CO₂-eq, 386.5 MJ and 8.74 m^3^ world eq for the weighted treatment mix. Annual totals were 4,479 kg CO₂-eq for HD and 4,482 kg CO₂-eq for OLHDF, a small difference per patient year. This small gap arose from the additional 27 L of purified water used per OLHDF session to generate substitution fluid.

The magnitude of the dialysis footprint observed here aligns with studies in other high-income settings. In Australia, HD was estimated to generate 4–6 tonnes CO₂-eq per patient annually [16], while UK data reported similar figures when including water and procurement 18. Our results are also consistent with earlier pathway-based analyses in Italy, where session operations and water systems were identified as hotspots [15]. These consistencies strengthen the external validity of our findings and suggest that treatment setting and system organisation, including whether patient and staff travel are included within system boundaries, play a major role in shaping reported footprints. Differences in transport patterns, electricity grids and boundary choices across studies mean that cross-country comparisons should be interpreted cautiously.

PD provides a useful comparator. A recent PD pathway LCA showed lower energy use but higher reliance on plastics, with procurement and waste dominating environmental burdens [14]. By contrast, HD and OLHDF impacts are dominated by patient and staff travel, followed by materials and devices, with machine electricity and water production contributing a smaller share in this centre’s context. These modality specific differences underscore the need for tailored sustainability strategies in kidney care.

The additional water requirement for OLHDF is consistent with prior reports. Maduell et al. documented a higher fluid demand for OLHDF compared with HD across European centres 19. Minor increases in energy consumption have been reported for OLHDF systems, though these have yet to be quantified, reflecting the extra loads required for convection and substitution fluid generation [4, 27, 18],. From a nephrology standpoint, these operational differences mirror the physiological advantage of OLHDF in convective clearance, achieved through higher filtration volumes.

The CONVINCE trial demonstrated that high-dose OLHDF improved survival compared with high-flux HD, supporting the clinical superiority of OLHDF [3]. A cost–utility analysis alongside the trial reported gains in quality-adjusted life years but higher overall costs due to longer survival and increased healthcare utilisation [17]. From an environmental perspective, the difference between modalities over a single treatment year was small. However, considering the longer average patient survival and reduced complication rates associated with OLHDF, an alternative functional unit based on lifetime treatment rather than 1 year could alter the relative interpretation. Over a full patient lifetime, cumulative environmental impacts for OLHDF would be higher because of extended treatment duration, even if per-session efficiency remains similar. This distinction highlights the importance of aligning environmental comparisons with clinically meaningful time horizons.

Opportunities to reduce the environmental footprint of dialysis were identified across operational, technical and systemic aspects of care:

- Water system efficiency. OLHDF requires approximately 27 L more ultrapure water per session than HD due to substitution fluid generation. Some RO systems typically reject 40–60% of input water **[19]**. Increasing recovery efficiency to 70–80%, as already implemented at the Modena centre, can reduce feed and reject water volumes by about 30%, equivalent to tens of thousands of litres saved per patient per year. Reuse of RO reject water for cleaning or irrigation has been successfully demonstrated **[20]**. Such measures can be integrated into existing dialysis unit operations without affecting patient throughput or safety.
- Energy reduction. Measured electricity use for OLHDF was 5.7% higher than for HD, mainly due to the substitution pump and convective flow. Optimising machine standby modes, scheduling sessions to flatten demand peaks and integrating renewable energy into dialysis units could reduce emissions [22]. Heat recovery from RO reject water is another underexplored opportunity [23].
- Procurement improvements. OLHDF uses a dedicated tubing set with integrated substitution lines, leading to slightly higher plastic consumption. Previous LCAs have also shown that disposables contribute significantly to emissions [15]. Substituting lower-impact materials, reducing packaging and piloting reuse models (e.g., dialyser reprocessing) could help, provided patient safety is maintained [24].
- Waste management. The higher material input for OLHDF produces slightly greater waste volumes. Innovations such as pyrolysis of dialysis plastics could reduce emissions compared with incineration [25]. Improved segregation can also decrease hazardous waste volumes, reducing treatment requirements.
- Policy integration. Health systems such as the NHS have committed to net-zero emissions [9]. Dialysis services, as a high-impact specialty, should be prioritised for environmental reporting and targeted interventions within national sustainability plans [26].
- Travel reduction. With patient and staff travel accounting for almost three quarters of total impacts, interventions such as home dialysis, shared care closer to home, or low-emission transport could have the largest effect. Future scenario analyses could examine the impact of shifting patient and staff transport to low-emission vehicles and of sourcing electricity from low-carbon or 24/7 carbon-free supply contracts. These options were not modelled quantitatively in this study due to limited data on local implementation but represent important directions for further work.

The sensitivity analysis showed that increasing the share of OLHDF from 0 to 100% increased total annual emissions from 4,427 to 4,548 kg CO₂-eq, a small difference between modalities, indicating that modality choice had only a minor effect on overall impacts. Varying the annual number of sessions between 150 and 160 changed total results by less than 3%, consistent with the linear relationship between session frequency and resource use. External parameters had a greater influence: applying the EU-average electricity grid factor increased emissions by about 0.7% while improving RO recovery from 50 to 70% reduced feed and reject water volumes by approximately 30%, corresponding to savings of around 40,000 L per patient year and 3–4 kg CO₂-eq. A reduced-flow OLHDF scenario, based on a Qd/Qb ratio of 1.2 and 25 L convective volume, achieved a 25-L reduction in water use per session and a 3–4 kg CO₂-eq saving per patient year while maintaining comparable Kt/V. These results indicate that water system efficiency and electricity supply characteristics exert far greater influence on overall environmental outcomes than the proportion of HD and OLHDF treatments.

## Conclusion

This study assessed the environmental impacts of HD and OLHDF at a large Italian centre using life cycle assessment. The reconciled annual footprint was 4,469 kg CO₂-eq, 60,290 MJ and 1,364 m^3^ world-eq water per patient year. Patient and staff travel were the dominant contributors, together accounting for about 71% of total emissions, followed by the procurement of consumables. Machine electricity, water production and waste management contributed much smaller shares.

Environmental differences between HD and OLHDF were modest, with OLHDF producing 4,548 kg CO₂-eq per patient year compared with 4,427 kg CO₂-eq for HD. The higher footprint for OLHDF resulted from 5.7% greater electricity use, 27 L more ultrapure water per session and higher plastic use from the dedicated HDF tubing set. These small differences confirm that the environmental implications of modality choice are limited and that clinical decisions should continue to prioritise patient outcomes.

The findings highlight the greatest opportunities for improvement through travel reduction, procurement redesign, improved water recovery systems, renewable electricity integration and sustainable waste management. Changes in grid carbon intensity and RO recovery efficiency have a much greater influence on total impacts than the mix of HD and OLHDF treatments. Integrating these measures into kidney care pathways will be necessary to align dialysis services with national and international net-zero targets.

### Strengths and limitations

A key strength of this study is the use of primary data from a large clinical centre, allowing accurate representation of real operational conditions. Detailed process mapping and consistent life cycle modelling across both modalities provide a comprehensive and comparable assessment. The patient-year functional unit enables alignment with other dialysis LCAs, while reporting both LICA water scarcity results and measured operational water volumes improves transparency. Direct measurement of machine energy confirmed that OLHDF consumes approximately 5.7% more electricity than HD, while measured water use showed an additional 27 L per OLHDF session. Including these empirical data strengthens confidence in the small observed differences between modalities.

Both HD and OLHDF used two dialysate-side ultrafilters within the Fresenius 5008 machine, replaced quarterly in accordance with current clinical practice. This configuration minimises potential bias in comparing system performance and reflects modern operational standards.

Interpretation according to ISO 14044. In terms of completeness, the study includes the main life‑cycle stages for in‑centre HD and OLHDF (procurement, water treatment, session operations, travel and waste) while excluding pharmaceuticals, vascular access and infrastructure, which are expected to make a smaller contribution at the level of a single centre. Consistency was maintained by applying the same functional unit, system boundary and modelling assumptions across modalities and scenarios, and using Ecoinvent 3.11 as a common background database. Data quality is strongest for directly measured parameters (water, electricity, and material masses) and more uncertain for secondary data, such as travel distances and some waste flows. Parameter uncertainty, particularly in travel and RO performance, is partly explored through the sensitivity analysis; a full probabilistic uncertainty assessment was beyond the scope of this study and is highlighted as an area for future work.

Limitations include potential variation in equipment efficiency, infrastructure and clinical practices across dialysis centres, which may influence absolute results. The analysis was based on a single site and, therefore, reflects local electricity mix, treatment schedules and procurement sources. Despite these factors, the magnitude of the difference between HD and OLHDF was small, suggesting that moderate changes in input parameters would not alter the main conclusion that modality choice has minimal influence on overall environmental performance.

## Supplementary Information

Below is the link to the electronic supplementary material.

Supplementary file1 (DOCX 22 KB)

## Data Availability

Supplementary material is available through Zenodo. This includes the life cycle assessment dataset for HD in Modena (Larkin, J. 2024. 10.5281/zenodo.14259304) and product images for the HD/HDF dataset (Larkin, J. 2024. 10.5281/zenodo.14258920).
