# Supplementary material for: Comparing environmental footprints of haemodialysis and online haemodiafiltration in Italy
Source: Int Urol Nephrol. 2026 Feb 3;58(8):3305–15. doi: 10.1007/s11255-026-05033-3 (PMC13375663; doi:10.1007/s11255-026-05033-3)
Supplement: Supplementary file 1 — Supplementary file1 (DOCX 22 KB) [file 11255_2026_5033_MOESM1_ESM.docx]

### Appendix 1. Summary of primary measured data and secondary literature based parameters

### Table S1: Data sources for key inventory items

| **Inventory item** | **Data type** | **Description of parameter / use** | **Data source** | **Example Ecoinvent 3.11 process (background)** |
| --- | --- | --- | --- | --- |
| Dialyser mass and material | Primary (measured) | Mass and plastic type per dialyser; number per session | Modena procurement records; product dismantling and weighing ​ | manufacturing,  (region: Europe) ​ |
| Bloodline & tubing set | Primary (measured) | Mass and composition of HD vs OLHDF sets | Modena procurement; dismantling and weighing ​ | Same plastics / packaging processes as above ​ |
| Concentrate & saline volumes | Primary (measured) | Litres per session of acid, bicarbonate and saline | Nurse logs; stock usage records ​ | Production of chemical solution / NaCl in Ecoinvent ​ |
| Electricity – machine & RO | Primary (measured) | kWh per HD and OLHDF session (treatment + disinfection) | Plug‑in energy meter on Fresenius 5008 and RO units | “Electricity, medium voltage, IT, at grid” |
| RO product and reject water volumes | Primary (measured) | Litres of product and reject water per session | RO flow‑meter readings at Modena | “Tap water, at user, Europe” + wastewater treatment |
| Patient travel distance per session | Secondary (literature) | Average round‑trip km by car per HD session | Irish haemodialysis travel study | “Transport, passenger car, fleet average, Europe” |
| Staff commuting distance | Secondary (literature) | Average round‑trip km by car per HD session | Irish haemodialysis travel study | Same passenger car process as above |
| Waste treatment – hazardous clinical | Secondary (contractor + database) | Share autoclaved, incinerated, landfilled | Modena waste contractor documentation ​ | “Treatment of infectious waste, incineration” ​ |
| Waste treatment – packaging / general | Secondary (contractor + database) | Fraction to municipal waste / recycling | Modena waste contractor documentation ​ | “Municipal solid waste, treatment, sanitary landfill” ​ |
| Plastics and packaging production | Background (Ecoinvent) | Upstream production of polymer resins and packaging materials | Modelled only via Ecoinvent processes ​ | Polypropylene, granulate, at plant(Europe) ​ |
| Grid electricity mix | Background (Ecoinvent/EEA) | Italian grid emission factor (0.25 kg CO₂/kWh) | EEA GHG intensity; Ecoinvent electricity datasets ​ | “Electricity, medium voltage, production mix, IT” ​ |

### Appendix 2. Sensitivity analysis calculations

Baseline and constants

• Functional unit. One patient-year, 156 sessions.

• Baseline mix. 52% HD, 47% OLHDF.

• Totals used in Results. Baseline 4,469 kg CO2-eq, 60,290 MJ, 1,364 m³ world-eq.

• HD only. 4,427 kg CO2-eq, 58,900 MJ, 1,364 m³.

• OLHDF only. 4,548 kg CO2-eq, 62,250 MJ, 1,366 m³.

• Per-session carbon

– HD: 4,427 ÷ 156 = 28.378 kg CO2-eq

– OLHDF: 4,548 ÷ 156 = 29.154 kg CO2-eq

– Baseline mix: 4,469 ÷ 156 = 28.647 kg CO2-eq

• Measured electricity difference. OLHDF = 5.7% higher than HD. Energy difference 62,250 − 58,900 = 3,350 MJ per year, or 3,350 ÷ 156 = 21.5 MJ per session.

• Water difference. OLHDF uses 27 L more product water per session.

A. Modality share sweep

Definition. Compare HD-only vs OLHDF-only totals.

Calculation. ΔCO2-eq = 4,548 − 4,427 = 121 kg CO2-eq per patient-year.

% change vs HD-only = 121 ÷ 4,427 × 100 = 2.7%.

Interpretation. Moving from 0 to 100% OLHDF raises annual carbon by about 2.7%.

B. Session frequency

Scenarios provided: 150 and 160 sessions per year, HD-only.

Calculations vs baseline 4,469 kg CO2-eq

• 150 sessions: 4,270 kg, change = −199 kg, % = −199 ÷ 4,469 × 100 = −4.45 %.

• 160 sessions: 4,555 kg, change = +86 kg, % = +86 ÷ 4,469 × 100 = +1.92 %.

Calculations vs HD-only 4,427 kg CO2-eq

• 150 sessions: change = −157 kg, % = −157 ÷ 4,427 × 100 = −3.55 %.

• 160 sessions: change = +128 kg, % = +128 ÷ 4,427 × 100 = +2.89 %.

C. Electricity grid factor

Scenario. EU-average grid factor raises total to 4,500 kg CO2-eq.

Calculation. Change = 4,500 − 4,469 = +31 kg CO2-eq.

% = 31 ÷ 4,469 × 100 = 0.69 %.

D. RO recovery efficiency

Assumptions

• Product water per session. HD 382 L, OLHDF 409 L.

• Annual product water

– HD: 382 × 156 = 59,592 L

– OLHDF: 409 × 156 = 63,804 L

• RO recovery r. Feed water = product ÷ r. Reject = feed − product.

Feed and reject volumes

• At 65 % recovery

– HD: feed 59,592 ÷ 0.65 = 91,680 L, reject 32,088 L

– OLHDF: feed 63,804 ÷ 0.65 = 98,160 L, reject 34,356 L

• At 50 % recovery

– HD: feed 119,184 L, reject 59,592 L

– OLHDF: feed 127,608 L, reject 63,804 L

• At 70 % recovery

– HD: feed 59,592 ÷ 0.70 = 85,131 L, reject 25,539 L

– OLHDF: feed 63,804 ÷ 0.70 = 91,149 L, reject 27,345 L

Water-related carbon saving estimate

Method. Derive a product-water emission factor from HD water production.

• HD water production carbon = 32 kg CO2-eq per year.

• Emission factor per litre of product water = 32 ÷ 59,592 = 5.37×10⁻⁴ kg CO2-eq per L.

• Use this factor to estimate changes when product-water use falls.

E. Reduced-flow OLHDF scenario

Prescription. Same adequacy, Qd reduced so dialysate falls by 25 L per session, convective 25 L maintained.

Water saving. 25 L per session × 156 = 3,900 L per year.

Carbon saving from water production. 3,900 × 5.37×10⁻⁴ = 2.09 kg CO2-eq per year.

Note. Additional savings from lower pumping may add about 1 kg CO2-eq, giving the 3 to 4 kg CO2-eq range reported.

F. Electricity difference per session and %

Given energy totals

• HD energy = 58,900 MJ per year.

• OLHDF energy = 62,250 MJ per year.

Difference = 3,350 MJ per year.

Per session difference = 3,350 ÷ 156 = 21.5 MJ per session.

% difference vs HD = 3,350 ÷ 58,900 × 100 = 5.69 %.

G. Summary table for Appendix

Scenario, Carbon kg CO2-eq per year, Energy MJ per year, LCIA Water m³ world-eq per year, Change vs baseline carbon

Baseline mix 52 % HD and 47 % OLHDF, 4,469, 60,290, 1,364, reference

HD only 0% OLHDF, 4,427, 58,900, 1,364, −42 kg, −0.94 %

OLHDF only 100%, 4,548, 62,250, 1,366, +79 kg, +1.77 %

Modality swing 0 to 100 %, +121 kg, +2.73 % vs HD-only, as above

150 sessions, 4,270, 57,940, 1,312, -199 kg vs baseline, -4.45 %

160 sessions, 4,555, 61,730, 1,384, +86 kg vs baseline, +1.92 %

EU grid factor 0.29 kg CO2 per kWh, 4,500, n.a., n.a., +31 kg, +0.69 %

RO recovery 50 %, n.a., n.a., HD feed 119 m³ reject 59.6 m³, OLHDF feed 128 m³ reject 63.8 m³, volumetric only

RO recovery 70 %, n.a., n.a., HD feed 85.1 m³ reject 25.5 m³, OLHDF feed 91.1 m³ reject 27.3 m³, volumetric only

Reduced-flow OLHDF minus 25 L dialysate per session, 4,515, 60,290, 1,339, about −2 to -4 kg vs standard OLHDF for water production alone

H. Notes to replicate calculations

1. Per-session carbon values use total ÷ 156 sessions.
2. % changes use scenario minus comparator, divided by comparator.
3. RO volumes use feed = product ÷ recovery, reject = feed − product.
4. Water-production carbon factor derived from HD water production value and product-water litres.
